# An open-source deep learning-based toolbox for automated auditory brainstem response analyses (ABRA)

**DOI:** 10.1038/s41598-026-38045-1

**Published:** 2026-02-19

**Authors:** Abhijeeth Erra, Cayla M. Miller, Jeffrey Chen, Elena Chrysostomou, Shannon Barret, Yasmin M. Kassim, Rick A. Friedman, Amanda Lauer, Federico Ceriani, Walter Marcotti, Cody Carroll, Uri Manor

**Affiliations:** 1 Data Institute, University of San Francisco, San Francisco, CA USA; 2 Department of Cell & Developmental Biology, University of California San Diego, La Jolla, CA USA; 3 Department of Otolaryngology, University of California San Diego, La Jolla, CA USA; 4 Departments of Otolaryngology-Head and Neck Surgery and Neuroscience, Center for Functional Anatomy and Evolution, Johns Hopkins University School of Medicine, Baltimore, MD USA; 5 School of Biosciences, University of Sheffield, Sheffield, S10 2TN UK; 6 Neuroscience Institute, University of Sheffield, Sheffield, S10 2TN UK; 7 Department of Mathematics and Statistics, University of San Francisco, San Francisco, CA USA; 8 Halıcıoğlu Data Science Institute, University of California San Diego, La Jolla, CA USA

**Keywords:** Auditory system, Electrophysiology

## Abstract

Hearing loss is a pervasive global health challenge with profound impacts on communication, cognitive function, and quality of life. Recent studies have established age-related hearing loss as a significant risk factor for dementia, highlighting the importance of hearing loss research. Auditory brainstem responses (ABRs), which are electrophysiological recordings of acoustically evoked synchronized neural activity from the auditory nerve and brainstem, serve as in vivo correlates for sensory hair cell and synaptic function, hearing sensitivity, and other critical readouts of auditory pathway physiology, making them highly valuable for both basic neuroscience and clinical research. Despite the utility of the ABR, traditional ABR analyses rely heavily on subjective manual interpretation, which may introduce variability and pose challenges for reproducibility across studies. Here, we introduce Auditory Brainstem Response Analyzer (ABRA), a novel suite of open-source ABR analysis tools powered by deep learning, which automates and standardizes ABR waveform analysis. ABRA employs convolutional neural networks trained on diverse datasets collected from multiple experimental settings, achieving rapid and unbiased extraction of key ABR metrics, including peak amplitude, latency, and auditory threshold estimates. We demonstrate that ABRA’s deep learning models provide performance comparable to expert human annotators while dramatically reducing analysis time and enhancing reproducibility across datasets from different laboratories. By bridging hearing research, sensory neuroscience, and advanced computational techniques, ABRA facilitates broader interdisciplinary insights into auditory function. An online version of the tool is available for use at no cost at https://abra.ucsd.edu.

**Supplementary Information:**

The online version contains supplementary material available at 10.1038/s41598-026-38045-1.

## Introduction

 Hearing loss is a prevalent and debilitating condition affecting hundreds of millions worldwide. In addition to significantly diminishing quality of life, age-related hearing loss has emerged as a major risk factor for cognitive decline and dementia, underscoring the urgent need for better research tools and treatment strategies. Both age-related hearing loss and dementia involve the progressive loss of synapses (synaptopathy) - in the cochlea and brain, respectively - highlighting common neurodegenerative mechanisms that warrant intensive study^1–7^.

**Fig. 1:**
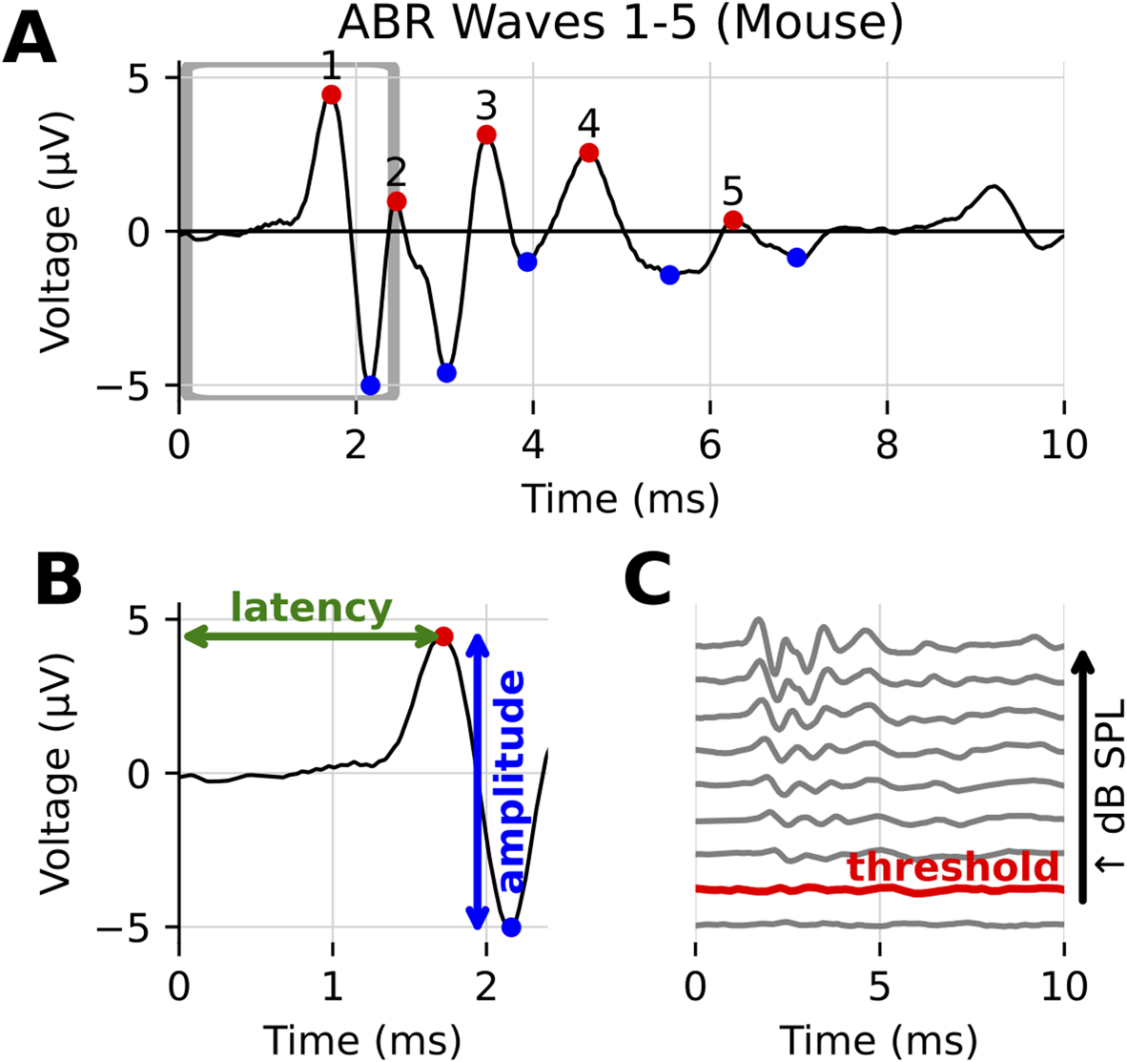
Example of ABR waveforms recorded from a mouse, showing its characteristic features. (**A**) One 10 ms ABR recording, with the five characteristic peaks denoted by red dots, and their corresponding troughs with blue dots. (**B**) A close up of the boxed region in (**A**), showing how latency (time to peak) and amplitude (peak to trough height) are defined for wave 1. (**C**) Several ABR recordings at varying sound levels, for the same mouse and frequency. The threshold level above which the ABR response is indicative of hearing is shown in red.

Among the most powerful approaches to assess auditory function are auditory brainstem response (ABR) recordings, which objectively measure electrical activity along the auditory neural pathway, from cochlear inner hair cells through the brainstem^8–13^. In mice, ABRs consist of five characteristic peaks approximately corresponding to neural signals propagating through sequential auditory structures, though some centrally generated waves may reflect concurrent activity in multiple structures (Fig. 1)^14–17^. Physiological and mathematical models of ABR morphology have also been developed^18^.

Two particularly informative measurements obtained from ABRs—hearing threshold sensitivity and wave 1 characteristics—have been shown to correlate strongly with cochlear-based hearing damage; specifically, ABR thresholds and wave 1 amplitudes are affected by changes in cochlear function such as hair cell loss or cochlear synaptopathy^2,19–21^. However, current ABR analysis methods typically rely on manual waveform interpretation, which can be subjective, labor-intensive, and prone to inconsistency between individual researchers or labs^22,23^.

Heuristic and machine learning computational approaches have been explored for automated ABR analysis^24,25^. Early methods focused on hand-engineered features (e.g., manually calculating waveform curvature)^20^ and statistical classifiers, such as support vector machines for threshold detection^26^ or model-based approaches for identifying near-threshold responses^27^. Supervised learning models (i.e. models which learn from data with ground truth labels) like convolutional neural networks (CNNs), gradient boosting machines, and others have been used to accurately analyze suprathreshold ABR waveforms^28,29^ and to assess the degree of synaptopathy in humans^30^.

In this paper, we introduce the Auditory Brainstem Response Analyzer (ABRA), a collection of novel open-source tools, including machine learning models trained on a diverse set of mouse data ABR to enable comprehensive and maximally generalizable mouse ABR analysis. Two algorithms are provided to (1) automatically estimate thresholds and (2) detect peaks and quantify latencies. To make these algorithms broadly accessible, we have packaged them into a user-friendly API and accompanying browser-based application that also supports batch data import/export, waveform visualization, and interactive 2D/3D plotting. By integrating these diverse functionalities into a unified platform, ABRA aims to streamline ABR data processing and analysis, reduce manual labor, and facilitate standardization and reproducibility across labs. We demonstrate the flexibility and generalizability of these algorithms by benchmarking the performance on ABR datasets collected from three different hearing research labs using distinct experimental protocols and recording settings.

## Methods

### Data collection

To test the generalizability and flexibility of the open-source ABR software developed here (ABRA v0.1.1; https://abra.ucsd.edu^31^), we trained and tested the models using three distinct ABR datasets from separate laboratories (Table 1). While the three laboratories used a similar overarching methodology, each used unique experimental protocols, including varying collection software, sound source and stimulus frequencies, and mouse strains, including mouse models of accelerated aging and mice exposed to temporary threshold shift-inducing noise. These differences underscore the flexibility of ABRA in accommodating diverse experimental setups and protocols. Further details on data collection conditions are available in the Supplementary Information (Supplementary Table S1).

**Table 1:** Details of mouse and individual ABR waveforms used from the three different laboratories (Lab A, lab B, lab C) in the model split into a train and test datasets. Figures and tables relevant to a given dataset are enumerated in brackets in the last row.

Lab/Model	Peak Detection		Automatic Thresholding			
	Training Data	Test Data	Training Data	Test Data	Interrater Comparison	ABRA vs. EPL-ABR
Lab A	32 mice (288 ABRs)	8 mice (80 ABRs)	35 mice (5,277 ABRs)	9 mice (1,393 ABRs)	–	–
Lab B	83 mice (8,635 ABRs)	21 mice (2,247 ABRs)	75 mice (11,700 ABRs)	19 mice (2,966 ABRs)	10 mice (1,560 ABRs)	–
Lab C	–	–	95 mice (5,665 ABRs)	24 mice (1,513 ABRs)	–	2 mice (122 ABRs)
Total [Relevant Figures & Tables]	115 mice (8,923 ABRs) [Fig. 2**]**	29 mice (2,327 ABRs) [Figs. 4 and 5; Table 2**]**	205 mice (22,642 ABRs) [Fig. 3**]**	52 mice (5,872 ABRs) [Fig. 6; Table 3**]**	10 mice (1,560 ABRs) [Fig. 7; Table 4**]**	2 mice (122 ABRs) [Table 5**]**

### Ethics and study design

ABR datasets from two contributing laboratories have been previously published (Lab B:^32^; Lab C:^33)^ and were collected in accordance with the ethical approvals described in those studies. Additional recordings from the Manor laboratory (Lab A) were obtained from available ABR data collected under ongoing studies; no new interventions were performed for the purpose of this work. These experiments were approved by the Institutional Animal Care and Use Committees (IACUC) at the Salk Institute for Biological Studies, protocol number 18–00052, and at the University of California, San Diego, protocol number S23058, and were performed in accordance with relevant guidelines and regulations.

For all datasets, the experimental unit was defined as the individual animal. For this study, animals were randomly selected for method development and testing as outlined in Table 1. Sample sizes were determined by data availability rather than statistical considerations, and all recordings with available human-generated ground truth data were included in the analysis. Data were analyzed in an automated manner without knowledge of experimental conditions or prior annotations.

All animal procedures are reported in accordance with the ARRIVE guidelines for the reporting of animal experiments (https://arriveguidelines.org). The focus of this study was development and validation of an automated ABR analysis pipeline rather than testing a biological hypothesis.

### ABR preprocessing

To provide consistent data into the downstream models for peak detection and thresholding, ABR waveforms from all labs were preprocessed to have the same sampling frequency and scale. First, each waveform stack (all waveforms for a particular frequency and mouse) was normalized so the minimum and maximum values span from 0 to 1. To place all ABRs on the same time scale, each waveform was provided to the CNN as a vector of length 244, covering 10 ms of recording. All data included in the model was recorded for at least 10 ms; longer recordings were truncated. Higher sampling frequencies were downsampled to 244 points using linear interpolation. While no data in our dataset had lower sampling frequencies, the provided code allows for lower sampling frequencies to be upsampled to 244 points using cubic spline interpolation. For the downsampled waveforms, we computed the power spectrum of the waveform to ensure the results were not affected by aliasing; we found on average 0.02% and at max 0.75% of the power was above the new Nyquist limit, making aliasing effects negligible.

### Peak detection

The ABRA toolbox incorporates a two-step peak finding algorithm: briefly, Convolutional Neural Network (CNN) predicts the location of the wave 1 peak, and then a second fine-tuning step improves this prediction and labels the remaining peaks.

The CNN was trained on ABR data from two labs (summarized in Table 1) labeled with ground truth peak 1 annotations. Ground truth labels from Lab A were hand-annotated by expert ABR practitioners; those for Lab B were labeled as previously described^32^. The training dataset of 8,923 ABRs from 115 mice (summarized in Table 1**)** was split into training and validation subsets, with 80%, (7,209 ABR) and 20% (1,714 waveforms) in each set, respectively. The loss contributions from each training sample were weighted to ensure that data from both labs were represented equally in the model training.

**Fig. 2:**
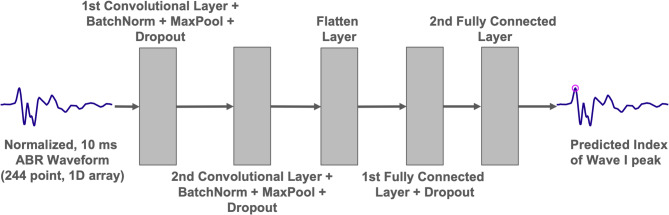
Model architecture for the wave 1 peak finding algorithm. The input (a 244-point ABR waveform spanning 10 ms) passes through two layers of convolution, batch normalization, max pooling (after ReLU activation), and dropout. The dimensionality of the output is then reduced through two consecutive fully connected layers using ReLU activation which returns the prediction of the time point of the wave 1 peak.

The CNN optimized squared error loss (L^2^) for the regression task which returns a prediction for the wave 1 peak index. The model architecture and training hyperparameters were determined by a randomized search over convolutional filter sizes, kernel sizes, dropout rates, pooling strategies, learning rates, weight decay values, and early stopping criteria. The validation set was evaluated at each training epoch, and if the validation loss did not decrease over 25 consecutive epochs, training was stopped to prevent overfitting. With this criterion, training stopped after 62 epochs. A simplified representation of the network architecture is shown in Fig. 2. The model hyperparameters chosen by cross-validation are displayed in Supplementary Table S2.

The CNN’s prediction of the wave 1 peak index was often close to, but not exactly at the local peak. An additional fine-tuning step was added to leverage this relatively accurate prediction with greater precision (Supplementary Figure S2). First, the waveform was smoothed using Gaussian smoothing (σ=1) to remove any noise which could cause spurious peak detection. Then the *find_peaks* method from Scikit-learn was used to identify all candidate peak and trough locations. The parameters for this function were optimized using ground truth wave 1 latency and wave 1 amplitude for the validation set and include the following:

- Waveform start point: 0.41 ms (10 sample points) before the CNN-predicted peak 1 location (earlier timepoints are not fed into *find_peaks*).
- Minimum allowed time between candidate peaks: 0.66 ms (16 sample points).
- Minimum time between candidate troughs: 0.29 ms (7 sample points).

Of these candidate peaks, our algorithm then identifies whether any are within ± 0.25 ms from the CNN prediction. If multiple peaks were present, the highest valued one was chosen. If no peaks were found within this window, the window was widened by an additional ± 0.25 ms until a peak was found. From the rest of the candidate peaks, the 4 peaks after this peak with the highest amplitudes (measured from 0) were chosen as peaks 2–5. From the candidate troughs, troughs 1–5 were chosen as the first trough that follows a given peak. The amplitudes corresponding to the identified peak and trough indices were quantified from the original (unsmoothed) waveforms.

### Supervised threshold estimation

The ABRA threshold estimation method includes two steps: First, a binary classifier labels individual ABR waveforms as either above or below threshold. Then, for a stack of waveforms at a given frequency, the threshold is defined as the lowest sound level predicted as hearing for two consecutive stimuli (e.g., if levels up to 30 dB are predicted as below threshold, 35 dB as above, 40 dB as below, and 45 dB and higher as above, the threshold is defined as 45 dB; Supplementary Figure S3). This method greatly increases generalizability across different datasets since it allows the model to estimate thresholds from inputs containing any number of waveforms. Three candidate supervised binary classifiers were trained and evaluated: A CNN classifier, an XGBoost classifier, and a Logistic Regression classifier.

Logistic Regression was selected as a baseline linear model because it allowed us to test whether simple linear combinations of waveform amplitudes across timepoints could be sufficient to classify the presence of a signal in the ABR. Its coefficients can provide direct information about which timepoints (i.e. regions around canonical peaks and troughs) contribute most strongly to classification, allowing for interpretability. XGBoost was chosen as a more complex model that could capture higher-order interactions between waveform features. It is known for its strong performance on tabular data. Together, these models provide a range of algorithm complexity against which the CNN’s performance can be compared.

These models were evaluated and compared using accuracy, true positive rate (TPR; i.e. the proportion of actual above threshold instances that are correctly identified as above threshold), false positive rate (FPR; i.e. the proportion of actual below threshold instances that are incorrectly classified as above threshold), the area under the receiver operator curve (AUC-ROC) and the area under the precision-recall curve (AUC-PR).

The dataset included 28,636 individual ABR waveforms from 259 mice (Table 1) across varying stimulus frequencies and sound levels. The ABRs were grouped by animal subject, then 80% of these waveform stacks from each lab were randomly allocated for training and the remaining 20% were designated for testing (see Table 1 for ABR counts from each lab). This minimized data leakage and ensured a representative distribution of ABRs from various subjects and labs across the training and testing sets.

Three models were evaluated for threshold estimation: Logistic Regression and XGBoost Classifiers, and a CNN. For the Logistic Regression and XGBoost Classifiers, time warping was applied to the ABR waveforms as an additional preprocessing step to align waveform features such as peaks and troughs (see Supplementary section: ABR Curve Alignment with Time Warping and Supplementary Figure S1). Because these models do not require a validation group, they were trained on the full training and validation set, a matrix with dimensions of 22,642 × 244, where 22,642 is the total number of training samples and 244 the number of voltage readings for each waveform. Unlike CNNs, these models do not learn hierarchical representations and are less able to benefit from augmentation techniques (described below) designed to teach invariances from raw signals.

To improve generalization and avoid overfitting, data augmentation techniques were used to increase the sample space used for training the CNN. This included noise injection, elastic augmentation through cubic spline interpolation, and time shifting. Noise augmentation added normally distributed noise scaled by 4% of the waveform magnitude. Elastic augmentation was applied by perturbing temporal indices with Gaussian noise (σ = 3) and resampling each sequence using cubic spline interpolation to produce smoothly distorted time axes. Time shifting was performed by randomly shifting each sequence up to ± 18 indices (0.73 ms) and padding the empty regions with Gaussian noise centered on the initial value (5% standard deviation). Thus, the final training input matrix for the CNN had dimensions of 36,096 × 244, including the 18,048 original training waveforms, and an augmented copy of each. An additional 4,594 waveforms were used as validation.

**Fig. 3:**
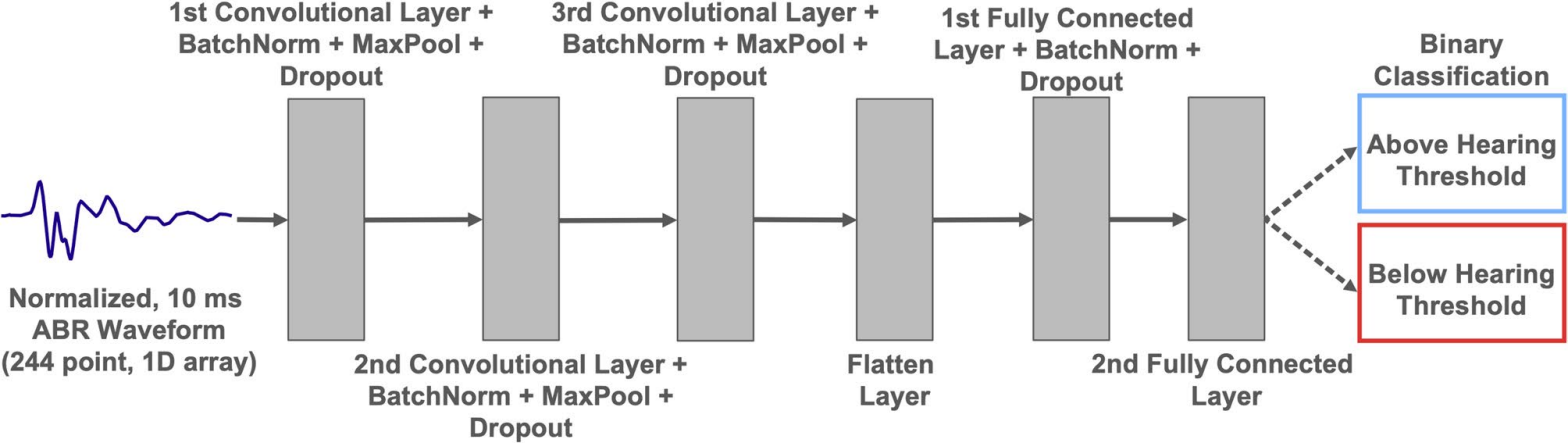
Model architecture for the CNN ABR threshold classifier. The input (a 244-point ABR waveform spanning 10 ms) passes through three sequential layers of convolution, batch normalization, max pooling (after ReLU activation), and dropout. The dimensionality of the output is reduced through two consecutive fully connected layers using ReLU activation before finally being passed through a sigmoid activation function returning the classification of the ABR.

As with the peak finding model, the final hyperparameters for the CNN architecture (shown in Fig. 3) were selected by randomized search to optimize model performance. The final selected hyperparameters are shown in Supplementary Table S2. Weighted loss was used to weight the loss contribution from each waveform, balancing the relative amount of data available from each lab.

## Results

### Peak amplitude and latency estimation

To benchmark the performance of the ABRA peak amplitude and latency estimation, we tested the performance on a set of 2,327 ABRs with human-labeled “ground truth” wave 1 amplitude and latency values from Lab A (80 waveforms from 8 mice) and Lab B (2,247 waveforms from 21 mice). The ground truth values for Lab A data were obtained by using visual examination with the BioSigRZ software from Tucker Davis Technologies (v5.7.6; https://www.tdt.com), while the ground truth values for Lab B data were obtained using a semi-automatic approach using custom software, as described in^32^. These ground truth annotations cover a wide range of tested frequencies and therefore latencies (Supplementary Figure S4). Though it is possible to make manual adjustments to these predictions, we assess the model here by comparing the automated (i.e. unadjusted) estimates from the ABRA peak finding algorithm vs. their corresponding human-labeled ground truth values.

**Fig. 4:**
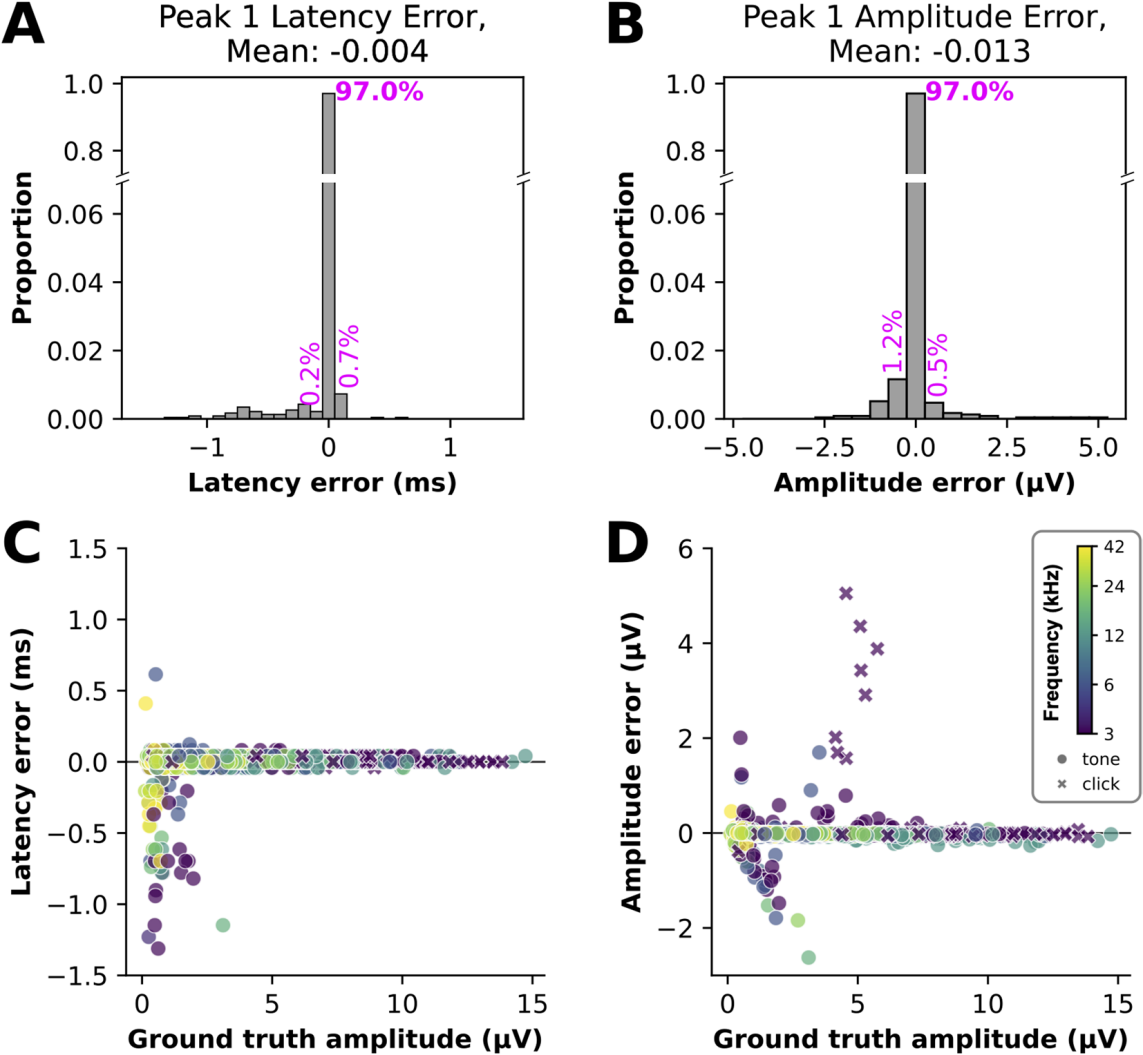
Errors in wave 1 peak quantification vs. ground truth for the ABRA peak finding method. (**A**) The distribution of errors in the predicted latency of the wave 1 peak (the direct output of the model). 97.0% of errors were within 0.05 ms of the ground truth measurement. (**B**) The distribution of errors in the wave 1 amplitude (peak to trough), calculated from the predicted wave 1 peak location, together with the estimated trough location. 97.0% of errors were within 0.25 µV of ground truth measurements. (**C**), (**D**). Wave 1 latency and amplitude errors vs. ground truth wave 1 amplitude, showing many of the highest errors occur on waveforms with small amplitudes (low SNR). Each predicted peak 1 is shown as a single semi-transparent point (circles for tone responses, x’s for click responses), color-coded by frequency. *n* = 2327 ABR waveforms in the test set. Related statistics are listed in Table 2.

**Table 2:** (Related to Fig. 5): table showing the mean error difference and their standard errors between ABRA-detected wave 1 latency and amplitude and corresponding ground truth values detected by human reviewers. Two sample t-tests found that mean error differences were not significant for wave 1 latency nor for wave 1 amplitude estimates at the 95% significance level after bonferroni correction.

		Wave 1 Latency (ms)	Wave 1 Amplitude (µV)
Mean Difference between prediction and ground truth (± S.E.M.)	Lab A (*n*_waveforms_=80, *n*_mice_=8)	-0.046 (± 0.021)	-0.115 (± 0.043)
	Lab B (*n*_waveforms_=2,247, *n*_mice_=21)	-0.003 (± 0.002)	-0.009 (± 0.005)
	Overall Test Set	-0.004 (± 0.002)	-0.013 (± 0.005)
RMSE (95% CI)	Overall Test Set	0.092 (0.088-0.095)	0.245(0.235-0.255)

**Fig. 5:**
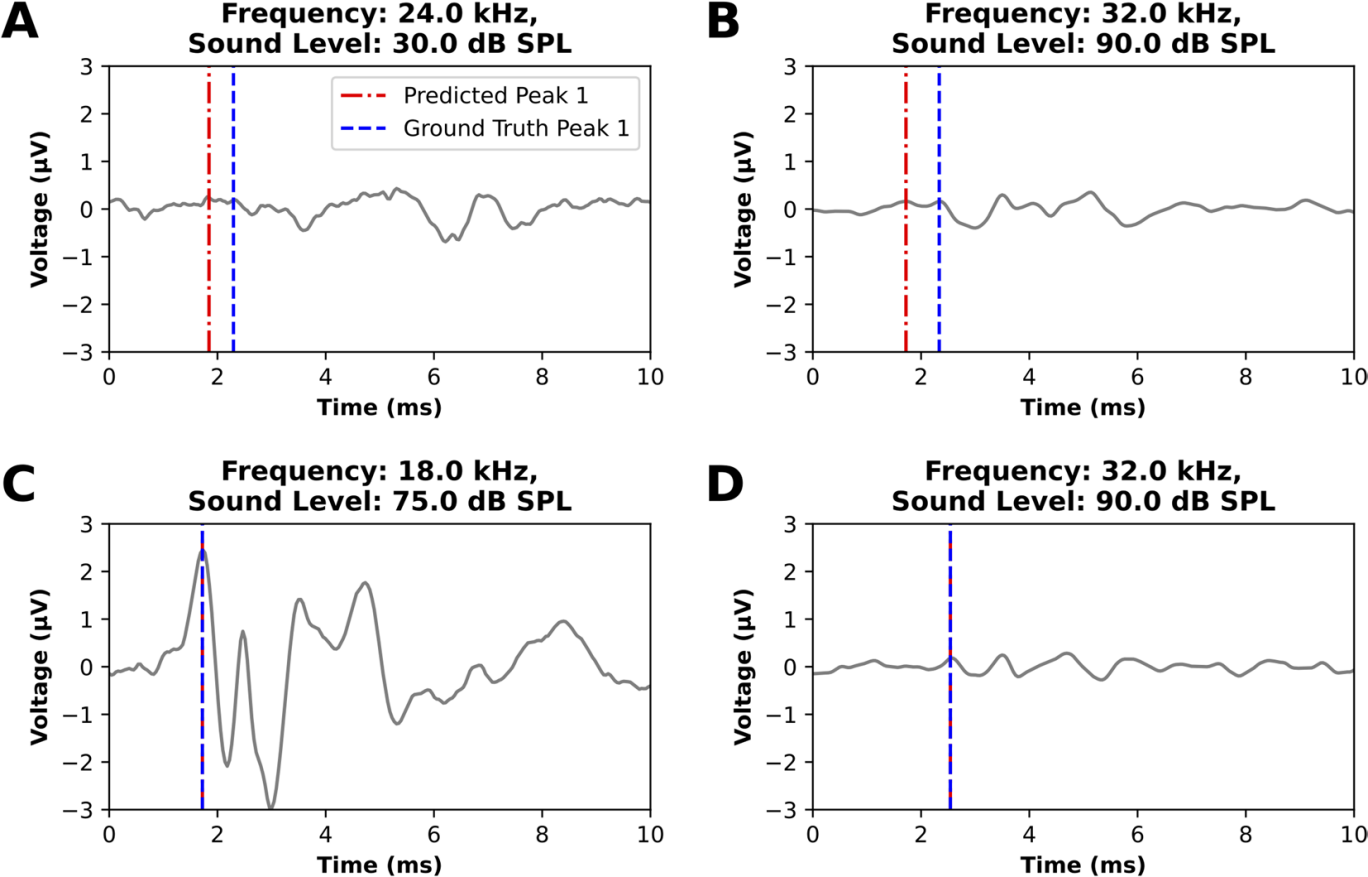
Examples of error and success cases in ABRA automated wave 1 peak detection. (**A**,** B**) For ABRs close to the ABR threshold and/or with low SNR, the ABRA peak detection algorithm sometimes identified the incorrect peak. (**C**) and (**D**) display examples of ABR waveforms with varying signal to noise ratios for which ABRA matched the ground truth.

Errors in the algorithm output (differences between the automated estimate and the human-annotated ground truth values) for the latencies and amplitudes of the wave 1 peak are shown in Fig. 4A and B, respectively; summary statistics for errors are reported in Table 2. Note that since many measurements had zero error, the average error of -0.004 ms is smaller in magnitude than the measurement precision of 0.041 ms (Table 2). While the root mean squared error in the peak latency was 0.092 ms, the mean absolute latency error was only *0.022* ms, or about 0.2% of the total sweep length. The low MAE shows that the prediction errors are generally small, while the over 4-fold greater RMSE is influenced by rare instances of larger error. These often occur in the low signal-to-noise ratio (SNR) regime (Fig. 4C). Randomly sampled examples of erroneous peak 1 predictions (Fig. 5A, B) occur in these low SNR waveforms; however, accurate predictions are also observed across a range of SNRs (Fig. 5C, D). Occasional amplitude errors at moderate amplitudes are often the result of misplaced trough 1 predictions (Fig. 4D, Supplementary Figure S5). The largest errors correspond to a small number of click responses from two animals and reflect ambiguity in trough placement rather than a systematic difference in performance between click and tone stimuli.

### ABR classification and threshold estimation results

**Fig. 6:**
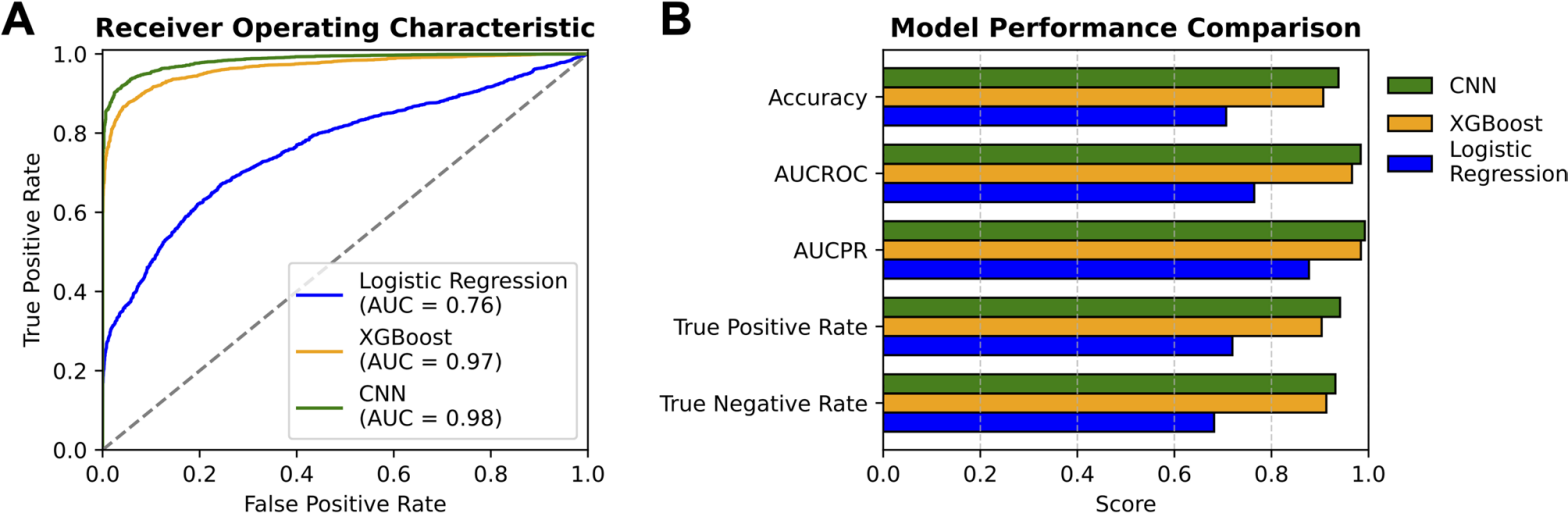
Comparison of machine learning models for ABR threshold classification. (**A**) Receiver Operating Characteristic (ROC) curves demonstrate the performance of each ABR classifier (top to bottom: CNN, XGBoost, and Logistic Regression) at all classification thresholds. The area under the ROC curve (Area Under Curve; AUC) represents each ABR classifier’s overall ability to distinguish between above-threshold and below-threshold ABR responses. (**B**) From top to bottom: Convolutional Neural Network (CNN), XGBoost, and Logistic Regression (baseline) are compared across the metrics Accuracy, Area Under the Receiver Operating Characteristic Curve (AUC-ROC), Area Under the Precision-Recall Curve (AUC-PR), True Positive Rate, and True Negative Rate. All metrics were weighted to represent each lab equally.

The performance of the three ABR classifiers for threshold detection was assessed on the testing set of 5,872 ABR waveforms. Performance metrics are shown in Fig. 6, and pairwise comparisons for these metrics are provided in Table 3. Logistic regression was used as a simple and interpretable baseline model for the binary classification task. However, its performance was significantly outperformed by the XGBoost model, which was further outperformed by the CNN across all metrics. The CNN model achieved an AUC-ROC of 0.98 and an AUC-PR of 0.99 (Fig. 6A), suggesting strong overall discrimination at different decision thresholds (AUC-ROC, Fig. 6B), excellent performance in handling class imbalance (AUC-PR), and reflecting robust sensitivity and precision.

**Table 3:** Comparative analysis of performance metrics between the three machine learning models for ABR threshold classification (related to Fig. 6). Metrics were calculated on the test set of 5,872 ABR waveforms, weighted to represent each lab equally, and compared between the convolutional neural network (CNN), XGBoost (XGB), and logistic regression (LR) models. The CNN model outperforms the XGB model across all metrics. Both CNN and XGB outperform the LR model across all metrics. P-values marked 0* are below the precision limit of 1E-300. Significance was calculated using two-sample proportion tests (Accuracy, TPR, FPR), delong’s test (AUC-ROC), or bootstrap resampling with 1000 iterations (AUC-PR). Standard errors were calculated from theoretical binomial variance (Accuracy, TPR, FPR), delong’s analytical variance (AUC-ROC) or from bootstrap resampling (AUC-PR). A bonferroni correction was applied to the p-values to correct for multiple comparisons. All metrics (Accuracy, AUC-ROC, etc.) and their difference estimates are reported as unitless proportions.

Metric	Comparison	Difference estimate (95% CI)	*p*-value
Accuracy	CNN vs. XGB	0.031 (0.022, 0.041)	10E-8
	CNN vs. LR	0.231 (0.218, 0.245)	10E-257
	XGB vs. LR	0.200 (0.186, 0.214)	10E-175
AUC-ROC Area Under the Receiver Operating Characteristic Curve	CNN vs. XGB	0.019 (0.016, 0.023)	10E-27
	CNN vs. LR	0.199 (0.187, 0.210)	10E-263
	XGB vs. LR	0.179 (0.168, 0.190)	10E-226
AUC-PR (Area Under the Precision-Recall Curve)	CNN vs. XGB	0.008 (0.008, 0.008)	0*
	CNN vs. LR	0.115 (0.115, 0.115)	0*
	XGB vs. LR	0.107 (0.107, 0.107)	0*
TPR (True Positive Rate)	CNN vs. XGB	0.038 (0.028, 0.048)	10E-12
	CNN vs. LR	0.222 (0.209, 0.235)	10E-244
	XGB vs. LR	0.184 (0.170, 0.198)	10E-149
TNR (True Negative Rate)	CNN vs. XGB	0.019 (0.009, 0.028)	10E-2
	CNN vs. LR	0.250 (0.236, 0.264)	10E-284
	XGB vs. LR	0.231 (0.217, 0.245)	10E-230

**Fig. 7:**
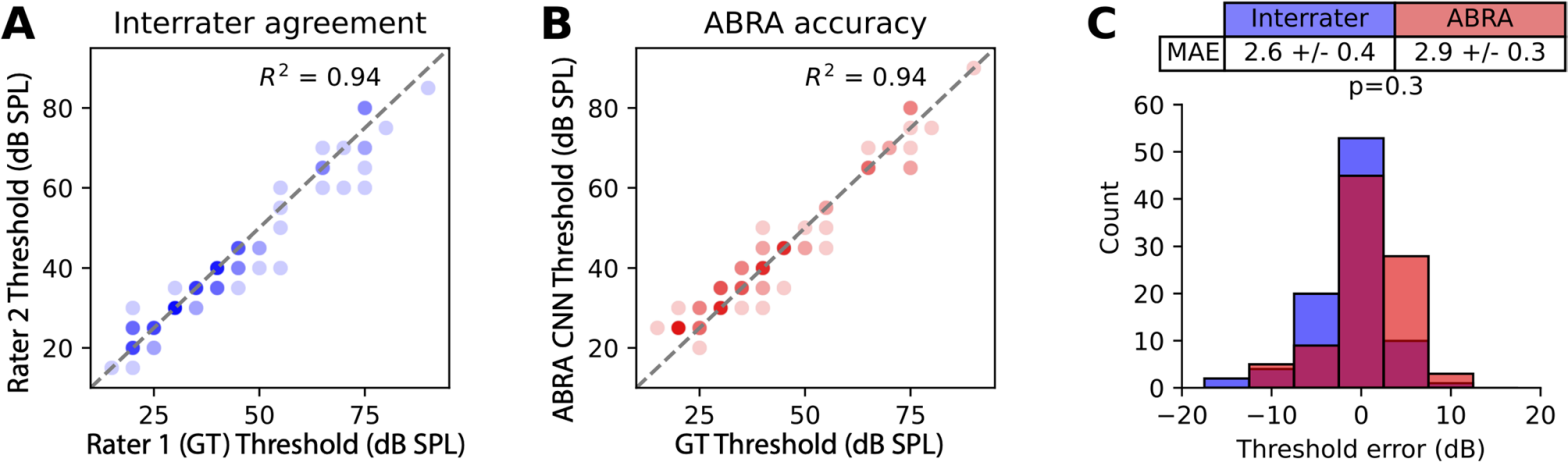
Threshold estimation accuracy of the ABRA thresholding algorithm and agreement among multiple expert raters. (**A**,** B**) Comparison of threshold estimates between ground truth (GT) rater and a second expert (**A**) and between GT and the CNN-based ABRA thresholding method (**B**), across 90 measurements. Each threshold estimate is displayed as a semitransparent point such that darker points represent multiple overlapping values. (**C**) Distribution of threshold errors for the same 90 measurements, with interrater differences in blue and ABRA thresholding errors in red. The absolute errors were not statistically different between interrater and ABRA comparisons (*p* = 0.34, t-test). The thresholds were estimated for 9 mice at 10 frequencies from Lab B. All data from Lab B was excluded from training and testing the CNN for this experiment.

To determine how much errors in our model may arise from variability in annotations by different researchers, used as the ground truth for training the model, we quantified the variability between expert annotators. We sampled 90 ABRs (10 mice at 9 frequencies) from Lab B and asked an expert from Lab A to independently determine the thresholds. The difference between the thresholds assigned by Rater 1 (Lab B) and Rater 2 (Lab A) represents the interrater error (Fig. 7A). We then trained the CNN on only data from Labs A and C, so that data from Rater 1 was not included in training. On average, the interrater error was comparable to that of the CNN on the sample of Lab B data (Fig. 7). While the mean absolute errors were similar, we also assessed the accuracy of the CNN predictions within 5 dB and 10 dB envelopes. At both the 5 dB and 10 dB cutoffs, the two were indistinguishable (Table 4). Together with the performance metrics above, this supports that the CNN model can function as a reliable tool for estimating hearing thresholds, providing a machine learning-based approach that matches human expert performance.

**Table 4:** Inference for differences in accuracy between inter-rater accuracy (IR) and the convolutional neural network (CNN) model in threshold estimation (related to Fig. 7). Within both the 5 and 10 dB SPL envelopes, no significant difference between the CNN baseline and inter-rater accuracy was detected, suggesting CNN is performing at a comparable level as a human reviewer at this precision.^22^

Accuracy envelope	CNN accuracy	IR accuracy	Accuracy difference (95% CI), CNN vs. IR	Bonferroni-corrected *p*-value
Within 5 dB SPL	0.91	0.92	-0.011 (-0.09, 0.07)	1.0
Within 10 dB SPL	1.0	0.98	0.022 (-0.01, 0.05)	0.3

The performance of our threshold estimation technique was compared against the cross-correlation algorithm embedded in EPL-ABR^22^ on a separate dataset of ABR waveforms from lab C (Table 5). This smaller set of ABR waveforms (N = 122) was selected because EPL-ABR’s threshold estimation software requires data in the custom ABR file format used by the Eaton Peabody laboratories (EPL). Our CNN method closely matches or outperforms EPL-ABR’s cross-correlation threshold estimation method across all metrics on this dataset.

**Table 5:** Comparison of the EPL-ABR cross-correlation algorithm and the ABRA CNN-based thresholding algorithm on lab C data (122 ABR waveforms from 2 mice). Reported metrics include Accuracy, True Positive Rate, False Positive Rate, and the ability to estimate thresholds within 5, 10, and 15dB SPL of the ground truth value. Values are presented as mean (± standard error). Standard errors for Accuracy, TPR, and FPR were computed from pooled binomial variance across all waveform classifications, while standard errors for the within-X dB metrics were computed as the sample standard error across the 12 threshold estimates.

Metric / Software	EPL-ABR cross-correlation^22^	ABRA Thresholding CNN
Accuracy	0.95 (± 0.02)	**0.98 (± 0.01)**
True positive rate	0.97 (± 0.02)	**1.0 (± 0.00)**
False positive rate	0.09 (± 0.05)	**0.06 (± 0.043)**
Within 5 dB SPL	0.92 (± 0.08)	**1.00 (± 0.00)**
Within 10 dB SPL	**1.00 (± 0.00)**	**1.00 (± 0.00)**
Within 15 dB SPL	**1.00 (± 0.00)**	**1.00 (± 0.00)**

Bold values indicate the best-performing method for each metric.

### Time cost analysis

In order to quantify the time savings of using the ABRA thresholding algorithm, a random sample of ABR files from 10 mice at 9 frequencies each for a total of 90 waveform stacks from Lab B was analyzed by two ABR raters from Lab A. It took both raters approximately 1 h to manually analyze the ABR thresholds. Using ABRA, it took about 48 s to output automated thresholds for all frequencies, corresponding to a 75x increase in efficiency. The automated thresholds were within 5 dB of Lab A inspection 90% of the time, within 10 dB 98% of the time, and within 15 dB 100% of the time. For comparison, inter-rater assessment showed that a Lab A annotator was within 5 dB of Lab B annotator’s result 92% of the time, 10 dB 98% of the time, and 15 dB 100% of the time.

**Fig. 8:**
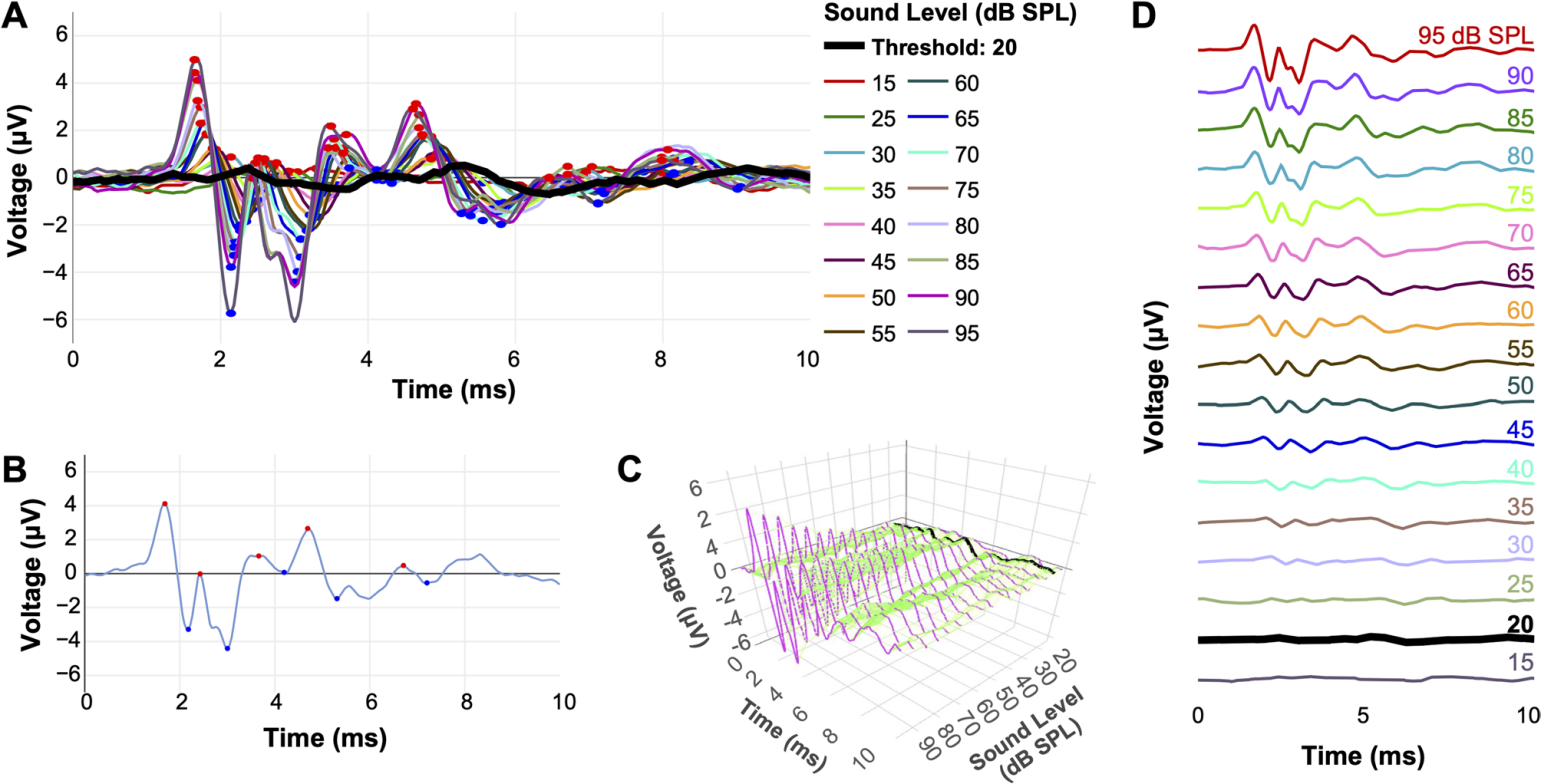
Visualizations of the combined ABRA thresholding and peak finding outputs. (**A**) Several ABR waveforms from a 1-month-old male C57Bl/6 N mouse across sound amplitudes at 18 kHz, with predicted peaks and troughs (red and blue circles, respectively) and the predicted threshold (thick black line). (**B**) A single waveform (18 kHz; 85 dB SPL) with peaks and troughs labeled. (**C**) 3D representation of the same ABR waveforms in (**A**), with the predicted threshold (20 dB SPL) in black, each waveform in pink and an approximation of the surface in green. (**D**) Stacked waveforms of the waveforms in (A), clearly showing the predicted threshold (20 dB SPL; black).

## Discussion

The deep learning techniques used in ABRA build on prior applications of machine learning to automate analyses of biological time-series data, including electrophysiology. Recent studies have shown that deep learning models such as convolutional neural networks and recurrent architectures can automatically extract meaningful features from complex, noisy biological signals and infer latent structure from high-dimensional recordings^34,35^. These efforts automate otherwise laborious and subjective analysis steps, enabling more accurate, reproducible, and time-efficient workflows. The deep learning techniques presented here have similar potential to streamline peak labeling and threshold identification in ABR studies. We further envision future ABR acquisition protocols in which data collection is adaptively guided by such algorithms to avoid unnecessary measurements once thresholds are reached. Together, this work contributes to the broader adoption of deep learning-based automation in electrophysiology analysis pipelines.

While ABRA is a powerful tool set for ABR analysis, like all existing ABR analysis programs, it also has limitations that highlight areas for future development. While the CNN-based predictions of peak location are powerful, they offer little transparency in terms of feature importance and interpretability. Future incorporation of interpretability methods may reveal waveform features that the model leverages to make accurate predictions across a large range of frequencies. Additional inclusion of biologically relevant priors in future versions may further reduce errors in peak finding. As for model limitations, these models were trained and validated only on mouse ABRs; future work could extend or train new deep learning models to handle non-murine ABRs, which may have reduced signal-to-noise due to larger distances from the source generators. Validation of automated amplitude and latency measurements has so far been restricted to wave 1, leaving waves 2–5 currently unvalidated; this can be pursued in future efforts as the model continues to incorporate new data from the labs mentioned here and others.

Another important consideration is the diversity of datasets used for model training: ABRA’s models have been trained on a diverse dataset including accelerated aging mouse models, mice with and without cadherin-23 correction, and two mouse lines exposed to varying noise exposures (Supplementary Table 1). However, this dataset is not exhaustive, and other datasets, especially those involving severe mutations, damage, or disease conditions may be significantly different from the training data. Such conditions, representing “out-of-distribution” cases, may require retraining or fine-tuning of the existing models. To address this challenge, transfer learning methods could be employed, facilitating rapid adaptation of existing deep learning models to new data conditions with minimal additional training data. Most importantly, the accuracy of peak and threshold detection may not yet match that of the most seasoned experts in visual ABR analysis for abnormal, ambiguous, or low signal-to-noise waveforms.

ABRA has been designed to be a multi-purpose and versatile suite of tools and accompanying web app with extended functionality to be able to handle datasets acquired from different mouse strains and experimental settings (Fig. 8; Supplementary Information: The ABRA Graphical User Interface). ABRA’s modular design allows its deep learning models to be used independently from the GUI, facilitating integration into other researchers’ computational workflows and software environments. It includes readers to facilitate processing of datasets recorded in different formats, including the widely used standard .arf files from BioSigRZ and BioSigRP Tucker Davis Technology recordings (v5.7.6; https://www.tdt.com), .tsv/.asc files from EPL’s Cochlear Function Test Suite (v1.0; CFTS software (Eaton-Peabody Laboratories v1.0; https://masseyeandear.org/research/otolaryngology/eaton-peabody-laboratories/engineering-core), or a generalized .csv file format from any number of other systems. ABRA’s automated thresholding method also reduces the time required for thresholding analyses by more than 50x compared to manual analysis and can streamline the process of extracting ABR thresholds from multiple subjects. All results can be exported to a .csv file for post-processing by the experimenter, and plots can be directly exported for publication if desired. While the time saved by automation alone may be a worthwhile tradeoff for certain applications, an additional benefit is the deterministic nature of the model and therefore high reproducibility. Most importantly, we anticipate significant improvements in performance as larger and more diverse datasets are incorporated over time.

In summary, ABRA represents a significant advancement in auditory neuroscience, merging cutting-edge deep learning technology with accessible and intuitive software design. By automating the analysis of auditory brainstem responses, a crucial in vivo indicator of auditory function, ABRA accelerates hearing research while enhancing reproducibility and consistency across diverse experimental settings. The accompanying user-friendly graphical interface, coupled with powerful computational models, makes advanced data processing accessible to researchers from various backgrounds, including neuroscientists, audiologists, and computational biologists. Beyond hearing research, ABRA exemplifies how artificial intelligence can effectively tackle complex biological data analysis, offering valuable insights into sensory neuroscience, neurodegenerative diseases, and beyond. Ultimately, ABRA provides a versatile and scalable solution, poised to facilitate transformative discoveries at the intersection of biology, medicine, and computer science.

## Supplementary Information

Below is the link to the electronic supplementary material.

Supplementary Material 1

## Data Availability

Data that support the findings of this study are publicly available from the following sources: Manor and Liberman Labs: [https://doi.org/10.5281/zenodo.15626375](https:/doi.org/10.5281/zenodo.15626375) ; Marcotti Lab: [https://doi.org/10.5281/zenodo.15619099](https:/doi.org/10.5281/zenodo.15619099) . Scripts are publicly available in the Github: [https://github.com/ucsdmanorlab/abranalysis](https:/github.com/ucsdmanorlab/abranalysis) .
