# Supplementary material for: An open-source deep learning-based toolbox for automated auditory brainstem response analyses (ABRA)
Source: Sci Rep. 2026 Feb 19;16:9855. doi: 10.1038/s41598-026-38045-1 (PMC13018588; doi:10.1038/s41598-026-38045-1)
Supplement: Supplementary file 1 — Supplementary Material 1 [file 41598_2026_38045_MOESM1_ESM.pdf]

Supplementary Information

Getting Started with ABRA

A tutorial manual for using the ABRA tools can be found on the tool's Github (https://github.com/ucsdmanorlab/abranalysis) and at the following link.

Details on Data Collection

The multi-lab dataset used here (summarized in **Table 1**), was collected from three separate labs, with data collection details shown in **Supplementary Table S1** below.

Methods	Lab A	Lab B	Lab C
**Anesthesia**	Ketamine (90 mg/kg) + Xylazine (10 mg/kg)	Ketamine (100 mg/kg) + Xylazine (10 mg/kg)	Ketamine (100 mg/kg) + Xylazine (10 mg/kg)
**Environment**	Soundproof chamber, heating pad (37°C)	Soundproof chamber, heating pad (37°C)	Soundproof chamber, heating pad (37°C)
**Electrode Placement**	Subcutaneous recording electrode at vertex, reference behind right pinna, ground on left leg	Subdermal electrodes behind pinna (reference and ground), vertex (active)	Needle electrodes: vertex to ipsilateral pinna (recording), ground near tail
**Sound Stimuli**	5-ms tone pips (0.5 ms cos2 rise-fall), 21/sec	5-ms tone pips (1.0-ms rise-fall with cos2 onset envelope), 42.6/sec; 0.01 ms clicks	5-ms tone pips (0.5-ms rise-fall with cos2 onset envelope), 30/sec
**Recording**	Filtered (300 Hz - 3 kHz), averaged using BioSigRZ software, 512 responses averaged	Customized software[1], RZ6 auditory processor, 256 responses averaged	Amplified (10,000X), filtered (100 Hz - 3 kHz), averaged with A-D board in LabVIEW system, 1024 responses averaged
**Recording sampling**	244 samples over 10 ms	1953 samples over 20 ms	426 samples over 17 ms
**Probed frequencies**	4, 8, 16, 24, and 32 kHz	Broad-range click; 3, 6, 12, 18, 24, 30, 36, and 42 kHz tones	8, 11.3, 16, 22.6, 32, and 45.2 kHz
**Sound Intensity**	Decreased from 90 dB SPL to 10/20 dB SPL in 5 dB steps	0-95 dB SPL (clicks); 15-95 dB SPL (tones) in 5 dB steps	Raised from ~10 dB SPL below threshold to 80 dB SPL in 5 dB steps
**Speaker distance**	Open-field - 10 cm from ear	Open-field - 10 cm from ear	Closed-field - ~3 cm from the eardrum
**Mouse age/strains used**	3-month SAMP8 (Senescence-Accelerated Mouse-Prone 8) [2]	1-month C57Bl/6N with and without corrected CDH23 [3]	7-week C57Bl/6J and CBA/CaJ, after varying noise exposures [4]

Supplementary Table S1: **Summary of the experimental recording conditions used by the three labs.** The specific methods employed by each lab—Manor Lab (Lab A), Marcotti Lab (Lab B), Liberman Lab (Lab C) in collecting each dataset are summarized, including anesthesia, environment, electrode placement, sound stimuli, response recording, sound frequencies and intensity, distance between the mouse and speaker, and mouse strains and ages.

The ABRA Graphical User Interface

The ABRA GUI incorporates the ABRA peak finding and thresholding tools and was developed in Python using the Streamlit framework [5], providing an interactive platform for researchers to visualize ABR data. All documentation of the code for the graphical user interface (GUI) and instructions for using the ABRA tools can

be found at: https://doi.org/10.5281/zenodo.15054979 [6]. The ABRA GUI allows users to import multiple ABR data files and accepts data in most commonly used formats: in .arf or exported .csv formats for data collected with the BioSigRZ/BioSigRP software (v5.7.6; https://www.tdt.com) and in .asc or .tsv format for data collected with the CFTS software (Eaton-Peabody Laboratories, v1.0; https://masseyeandear.org/research/otolaryngology/eaton-peabody-laboratories/engineering-core). For other data types, the application will accept any data converted into a generalized .csv format, and an example template is provided. Upon import, the data is preprocessed to extract the timescale of the recording, the stimulus frequencies and amplitudes, and the waveform data.

After import and preprocessing, the GUI allows the user to select which frequencies and sound levels they wish to examine for plotting and analysis. The ABR plots are shown through the Plotly framework in Python and can be downloaded as .png and .pdf files [7]. Calculated metrics related to the displayed waveforms are displayed under the plots, including wave 1 amplitude, latency to the first peak, and threshold (as defined in **Figure 1**). These metrics can be downloaded as a .csv file. The plotting functions allow the user to view all the waveforms for a single frequency, highlight the automatically detected peaks and troughs, and automate thresholding (**Figure 8**).

The ABRA interface also implements two novel visualization features for ABR waveforms of varying stimulus amplitudes at a given stimulation frequency. First, it provides the option to implement time warping, which visually aligns the peaks and troughs of multiple waveforms (see **Supplementary Figure S1**). This view does not change the underlying data, and does not affect wave amplitudes, but stretches and compresses the waveform to varying degrees along the time axis to enhance visualization. Second, the app provides an interactive 3D surface plot of waveforms which allows the user to view the series of ABR waveforms as a surface in the 3-dimensional space created by the time domain, the probed sound levels, and the recorded ABR voltage. These various functionalities can provide the user with tools to visually ascertain features like thresholds and peaks and automate model predictions of those features in tabular form. The ABRA tool set also provides automated batch analyses for multiple data files.

ABR Curve Alignment with Time Warping

ABRs from mice exhibit a characteristic structure with 5 distinct peaks (**Figure 1**). However, a common challenge in analyzing these ABR waveforms is the non-uniform latency across different frequencies and sound levels. This variability in latency can distort functional summary statistics (e.g. mean ABR curve, covariance surface) and time-based comparisons of these responses, as the peaks do not occur at the same time across different ABRs for the same mouse. To address this, we provide an option to employ time warping to align these ABRs, which aligns the position of peaks and other salient features of the ABRs across time. This alignment decouples amplitude from latency variation, facilitating the visual comparison of amplitudes of ABR waveforms. The encoding of time alignment parameters into individual-specific warping functions provides the option of incorporating these features into machine learning models, which in some cases improves the models' performance and predictive power as it did for the Logistic Regression and XGBoost Classifiers for automated thresholding. Because time warping adjusts the spacing between points on the time axis (without affecting the amplitude), the time warped curves can be used to visually inspect threshold or peak amplitude, but not latency, which should be assessed on the original unwarped curves.

To conduct the time warping step, we use the *fdasrsf* package in Python [8]. This package implements elastic time warping, a method that maximizes the alignment of key features in waveforms. Here, this technique provides aligned ABRs, counteracting the non-uniform latency across different frequencies and sound levels.

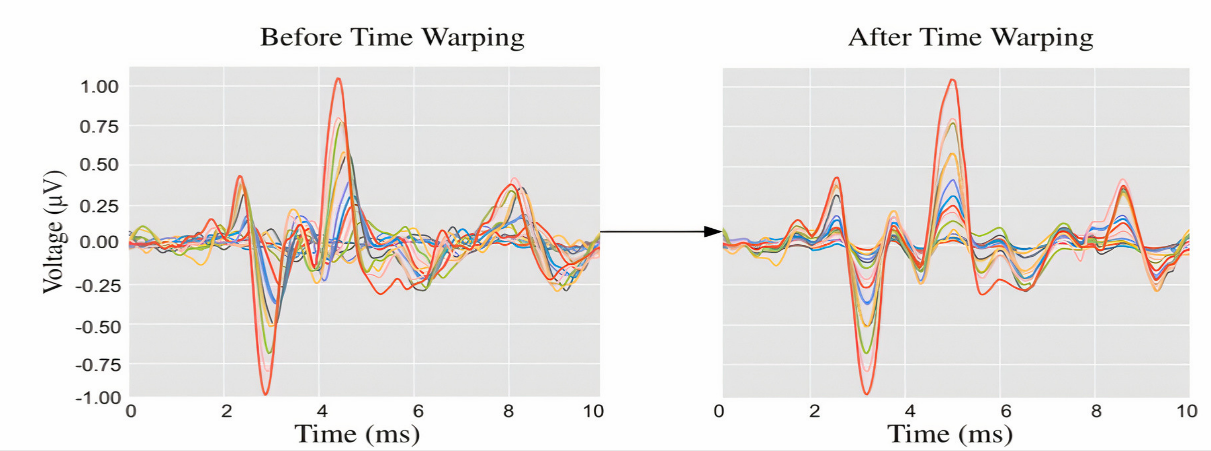

Supplementary Figure S1: ABRs before (left) and after (right) Time Warping. The depicted transformation of waveforms, both before and after applying elastic time warping using the *fdasrsf* package (Tucker 2021), illustrates clear registration of waveform features. Associated with each waveform is also an estimated time warping function which is useful in quantifying changes between the original unaligned latencies and the aligned latencies for all wave peaks and troughs.

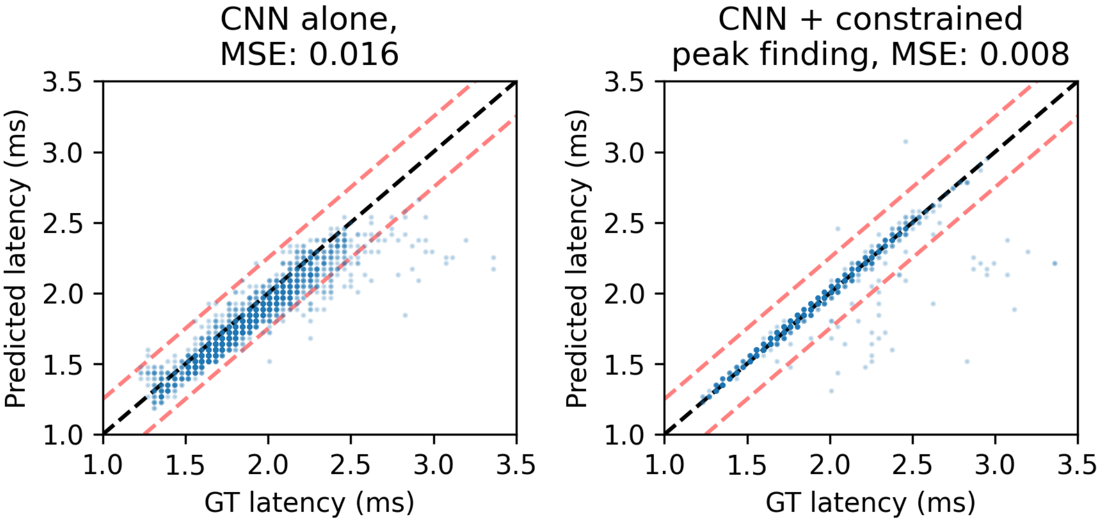

Supplementary Figure S2: Peak 1 detection, CNN alone vs. CNN with constrained peak finding. Predicted vs ground truth (GT) latency is shown for the direct output of the CNN (left) and the CNN output followed by the constrained peak finding function (right). Red dashed lines show +/- 0.25 ms error margins.

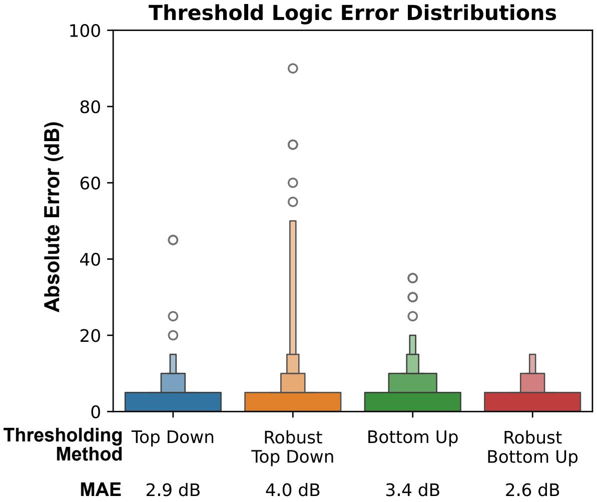

Supplementary Figure S3: Thresholding logic applied to the CNN predictions on the validation set. Four methods were tested. ***Top down:*** Working from high to low dB SPL, the first "below threshold" waveform is identified; the threshold is set as the previous ("above threshold") waveform. ***Robust top down:*** Similar to Top down, but requires two consecutive "below threshold" predictions.

Bottom up: The threshold is set as the first "above threshold" prediction when working from low to high dB SPL. ***Robust bottom up:*** Similar to Bottom up, but requires two consecutive "above threshold" predictions. Mean absolute error (MAE) for each method is shown below. Robust bottom up was used in all other figures and tables.

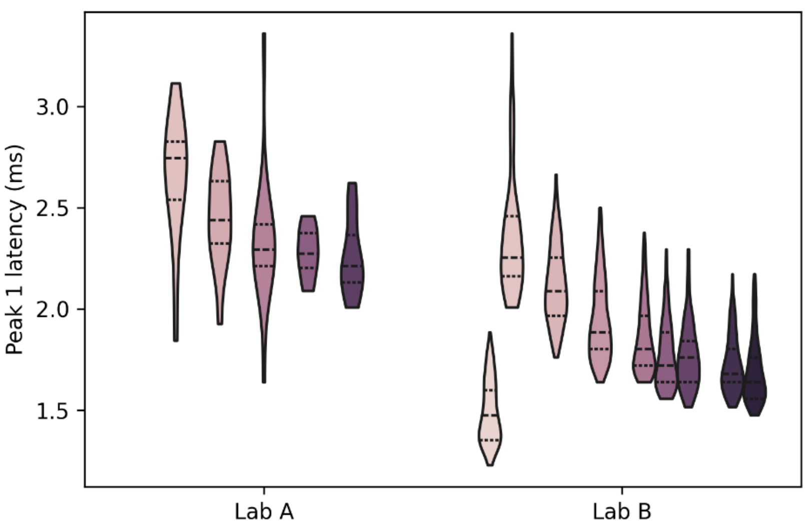

Supplementary Figure S4: Ground truth annotations for peak 1 latency. Annotations from Lab A include responses to 4, 8, 16, 24, and 32 kHz sound (left to right), and from Lab B include responses to Click, 3, 6, 12, 18, 24, 30, 36, 42 kHz sound (left to right).

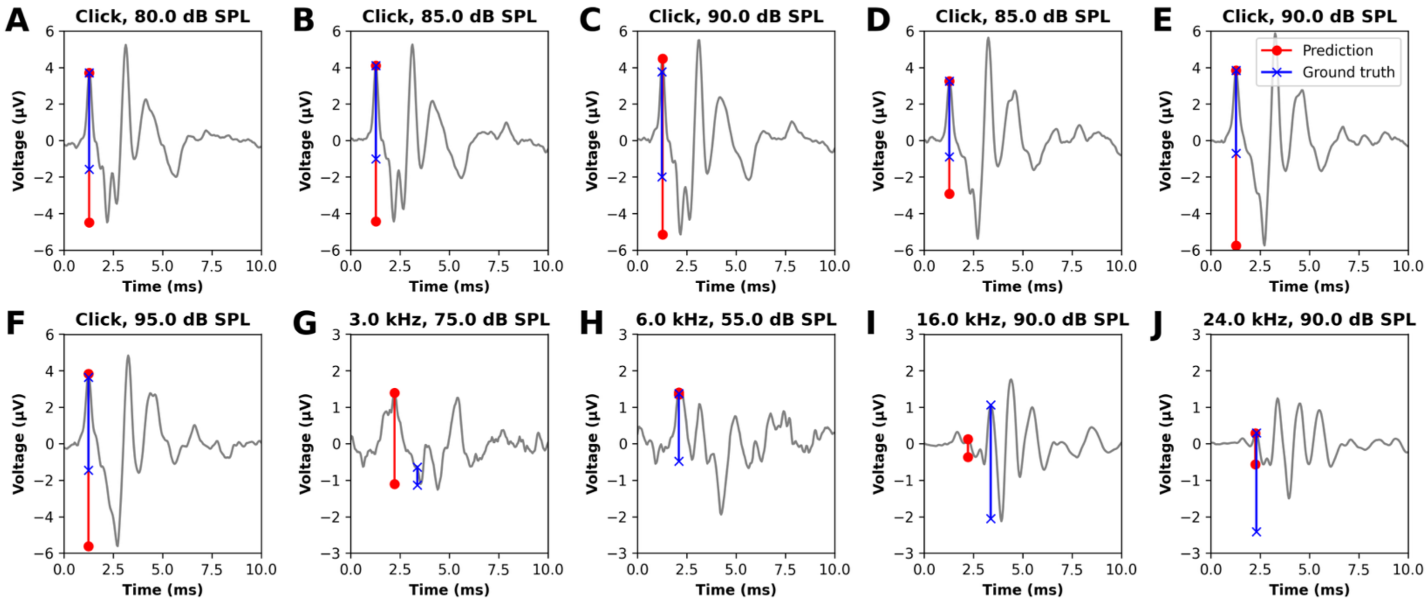

Supplementary Figure S5: The ten highest magnitude peak 1 amplitude errors. For each waveform, the predicted (red circles) and "ground truth" annotations (blue x's) are shown. The top point lies at the peak, and the line dropping down represents the peak amplitude. These top 10 errors come from a small number of cases: A, B, and C are Click responses from one mouse; D, E, and F are Click responses from a second mouse. H is an erroneously small amplitude due to a small trough near the top of peak 1. I and J are annotator errors.

ML Model Hyperparameters

Optimal choices of hyperparameters chosen by cross-validation for each of the thresholding and peak-finding models are displayed in **Table S2** below.

	Thresholding CNN	Thresholding XGB	Thresholding LR	Peak Finding CNN
Hyper-parameters	Loss function: Binary Cross Entropy<br>Activation function for all layers except final layer: Relu<br>Final activation function: Sigmoid<br>Batch Size: 128<br>Early Stopping Patience: 25<br>Reduce Learning Rate on Plateau Patience: 20<br>Optimizer: Adam<br>Learning Rate: 1e-4<br>Conv. Layer 1 Filters: 128<br>Conv. Layer 2 Filters: 128<br>Conv. Layer 3 Filters: 64<br>Conv. Layer Stride: 1<br>Conv. Layer Padding: 0<br>Kernel Size: 7<br>MaxPool Size: 2<br>MaxPool Stride: 2<br>MaxPool Padding: 0<br>Fully Connected Layer Size: 128<br>Dropout Rate between Conv. Layers and before first Fully Connected Layer: 0.5<br>Dropout Rate between Fully Connected Layers: 0.4	Subsample size for each tree: 1.0<br>Positive class weight: 1<br>Boosting iterations: 500<br>Minimum sum of instance weight (hessian) in a leaf node: 3<br>Maximum depth of a tree: 7<br>Learning Rate: 0.05<br>Gamma (Regularization parameter for tree splitting): 0.2<br>Fraction of features used for fitting each tree: 0.9<br>L1 regularization term: 1<br>L2 regularization term: 1	Scikit-learn defaults, Maximum iterations: 1000	Loss function: Mean Squared Error<br>Activation function for all layers: Relu<br>Batch Size: 32<br>Early Stopping Patience: 25<br>Optimizer: Adam<br>Learning Rate: 1e-3<br>Weight Decay: 1e-5<br>Conv. Layer 1 Filters: 128<br>Conv. Layer 2 Filters: 32<br>Conv. Layer Stride: 1<br>Conv. Layer Padding: 1<br>Kernel Size: 3<br>MaxPool Size: 2<br>MaxPool Stride: 2<br>MaxPool Padding: 0<br>Fully Connected Layer Size: 128<br>Dropout Rate between Conv. Layer 1 and Conv. Layer 2: 0.5<br>Dropout Rate between Conv. Layer 2 and first Fully Connected Layer: 0.3<br>Dropout Rate between Fully Connected Layers: 0.1
Library	Keras (3.3.3)	XGBoost (3.1.1)	Scikit-learn (1.7.2)	Pytorch (2.2.0.post100)

Supplementary Table S2: Candidate Model Hyperparameters for Thresholding and Peak Detection Models.

References

[1] N. J. Ingham, S. Pearson and K. P. Steel, "Using the auditory brainstem response (ABR) to determine sensitivity of hearing in mutant mice," *Current protocols in mouse biology,* vol. 1, p. 279–287, 2011.

[2] T. Takeda, M. Hosokawa, S. Takeshita, M. Irino, K. Higuchi, T. Matsushita, Y. Tomita, K. Yasuhira, H. Hamamoto, K. Shimizu and others, "A new murine model of accelerated senescence," *Mechanisms of ageing and development,* vol. 17, p. 183–194, 1981.

[3] F. Ceriani, J. Giles, N. J. Ingham, J.-Y. Jeng, M. A. Lewis, K. P. Steel, M. Arvaneh and W. Marcotti, "A machine-learning-based approach to predict early hallmarks of progressive hearing loss," *Hearing Research,* p. 109328, 2025.

[4] P.-z. Wu, L. D. Liberman and M. C. Liberman, "Noise-induced synaptic loss and its post-exposure recovery in CBA/CaJ vs. C57BL/6J mice," *Hearing research,* vol. 445, p. 108996, 2024.

[5] Streamlit., *A faster way to build and share data apps,* Snowflake Inc., 2025.

[6] A. Erra, C. M. Miller, J. Chen, Y. Kassim, L. Ashebir, P. Patel, C. Carroll and U. Manor, *ucsdmanorlab/abranalysis: ABRA v0.1.1 Release,* Zenodo, 2025.

[7] Plotly Technologies Inc., *Collaborative data science,* Montreal, QC: Plotly Technologies Inc., 2015.

[8] J. D. Tucker, *fdasrsf: Elastic Functional Data Analysis,* GitHub, 2025.
